# Zn- or Cu-containing CaP-Based Coatings Formed by Micro-Arc Oxidation on Titanium and Ti-40Nb Alloy: Part II—Wettability and Biological Performance

**DOI:** 10.3390/ma13194366

**Published:** 2020-09-30

**Authors:** Ekaterina G. Komarova, Yurii P. Sharkeev, Mariya B. Sedelnikova, Oleg Prymak, Matthias Epple, Larisa S. Litvinova, Valeria V. Shupletsova, Vladimir V. Malashchenko, Kristina A. Yurova, Anna N. Dzyuman, Irina V. Kulagina, Lyudmila S. Mushtovatova, Olga P. Bochkareva, Mariia R. Karpova, Igor A. Khlusov

**Affiliations:** 1 Laboratory of Physics of Nanostructured Biocomposites, Institute of Strength Physics and Materials Science SB RAS, 634055 Tomsk, Russia; sharkeev@ispms.tsc.ru (Y.P.S.); smasha5@yandex.ru (M.B.S.); 2 Research School of High-Energy Physics, National Research Tomsk Polytechnic University, 634050 Tomsk, Russia; 3 Inorganic Chemistry and Center for Nanointegration Duisburg-Essen (CeNIDE), University of Duisburg-Essen, 45141 Essen, Germany; oleg.prymak@uni-due.de (O.P.); matthias.epple@uni-due.de (M.E.); 4 Center for Immunology and Cell Biotechnology, Immanuel Kant Baltic Federal University, 236029 Kaliningrad, Russia; larisalitvinova@yandex.ru (L.S.L.); vshupletsova@mail.ru (V.V.S.); vvslon@rambler.ru (V.V.M.); kristina_kofanova@mail.ru (K.A.Y.); khlusov63@mail.ru (I.A.K.); 5 Department of Morphology and General Pathology, Siberian State Medical University, 634050 Tomsk, Russia; dzyman@mail.ru; 6 Department of Biochemistry and Molecular Biology, Siberian State Medical University, 634050 Tomsk, Russia; ikulagina@yandex.ru; 7 Department of Microbiology and Virology, Siberian State Medical University, 634050 Tomsk, Russia; mls2013@mail.ru (L.S.M.); bpo97@rambler.ru (O.P.B.); karpova.mr@ssmu.ru (M.R.K.); 8 Research School of Chemistry and Applied Biomedical Sciences, National Research Tomsk Polytechnic University, 634050 Tomsk, Russia

**Keywords:** micro-arc oxidation, calcium phosphate coating, Ti-40wt.%Nb alloy, wettability, biocompatibility, ectopic bone formation, bone lamellae, antibacterial efficacy

## Abstract

This work describes the wettability and biological performance of Zn- and Cu-containing CaP-based coatings prepared by micro-arc oxidation on pure titanium (Ti) and novel Ti-40Nb alloy. Good hydrophilic properties of all the coatings were demonstrated by the low contact angles with liquids, not exceeding 45°. An increase in the applied voltage led to an increase of the coating roughness and porosity, thereby reducing the contact angles to 6° with water and to 17° with glycerol. The free surface energy of 75 ± 3 mJ/m^2^ for all the coatings were determined. Polar component was calculated as the main component of surface energy, caused by the presence of strong polar PO_4_^3−^ and OH^−^ bonds. In vitro studies showed that low Cu and Zn amounts (~0.4 at.%) in the coatings promoted high motility of human adipose-derived multipotent mesenchymal stromal cells (hAMMSC) on the implant/cell interface and subsequent cell ability to differentiate into osteoblasts. In vivo study demonstrated 100% ectopic bone formation only on the surface of the CaP coating on Ti. The Zn- and Cu-containing CaP coatings on both substrates and the CaP coating on the Ti-40Nb alloy slightly decreased the incidence of ectopic osteogenesis down to 67%. The MAO coatings showed antibacterial efficacy against *Staphylococcus aureus* and can be arranged as follows: Zn-CaP/Ti > Cu-CaP/TiNb, Zn-CaP/TiNb > Cu-CaP/Ti.

## 1. Introduction

Titanium (Ti) and its alloys have been widely applied in orthopedics, traumatology, and dentistry from the last century due to their high chemical stability and excellent biocompatibility [1]. Despite their successful biomedical application, there are disadvantages and the most notable is high elastic modulus (~110 GPa) of α- and (α + β)-types Ti alloys [2]. High elastic modulus may result in stress shielding when unbalanced force distribution between bone and implant [3]. Recently, the β-type Ti alloys have drawn attention of scientists to the low elastic modulus of such alloys [2,4,5,6]. A novel binary β-type Ti-Nb-based alloys are promising materials for biomedical applications due to their relatively low elastic modulus, high corrosion resistance, and good biocompatibility [7,8]. These works [9,10,11] demonstrated that the elastic modulus of the Ti-Nb alloys varied in the wide range of 35–80 GPa depending on the Nb content. Kovalevskaya et al. [12,13] produced a Ti-40wt.%Nb (Ti-40Nb) alloy with elastic modulus of 70–80 GPa which can be used as material for orthopedic and dental implants.

Surface modification or functionalization is an efficient way to improve metal surface morphology, its chemical composition, and microstructure, which are important factors for implant biological performance. The studies [14,15,16] considered bioactive calcium phosphate (CaP) ceramic, similar in composition to bone tissue, which is applied on the Ti alloys to stimulate enhanced osseointegration and new bone formation. Micro-arc oxidation (MAO) also known as plasma electrolytic oxidation (PEO), is a novel method to prepare an in situ CaP coating with a wide range of physical and chemical properties on surface of valve metals and their alloys. MAO is considered to be a promising electrochemical method, allowing a synthesis of porous, rough, hydrophilic, and firmly adherent CaP coatings on metal surfaces and incorporation of other essential trace elements into the coatings [17,18,19,20]. The reviews [21,22,23,24] noted that the rough and porous CaP biocoatings can facilitate efficient osseointegration and biomechanical fixation due to the excellent bone cell adhesion and growth inside the microtopography on the coating surface. On the other hand, rough surface relief of CaP coatings, which is promising for osseointegration, can also provide unacceptable bacterial contamination [25]. Therefore, the development of both biocompatible and antibacterial CaP coatings, which provide excellent osseointegration and bacterial adhesion resistance, is a topical issue.

In recent years, modified MAO coatings with the incorporated metallic antibacterial ions (e.g., Zn^2+^, Cu^2+^ and Ag^+^) have become more appropriate due to the enhanced antibacterial properties in place of implantation and absence of cytotoxicity at low concentrations [20,26,27,28,29]. Such antibacterial coatings also promote delivery of therapeutics at particular areas inhibiting a development of bacteria biofilm as compared to an oral administration which has a higher risk of side effects and toxicity [21].

Influence of trace elements on the biological role of biocoatings has recently become an important topic in the research fields of bone formation and organism essential elements. Zinc (Zn) is involved in many metalloenzymes and proteins, including alkaline phosphatase (ALP) [20,28,29]. In vitro studies [29] demonstrated that Zn is an essential trace element, which promoted osteoblast adhesion, proliferation and differentiation, and inhibiting osteoclastic (bone-resorbing) activity. Hu et al. [20] showed in vitro that the adhesion, proliferation, and differentiation of rat bone marrow-derived multipotent mesenchymal stromal cells (BM-MMSC) on Zn-incorporated coatings were significantly enhanced compared with Zn-free coatings and commercially pure titanium plates. Furthermore, there is no cytotoxicity appeared on any of the Zn-incorporated coatings. In addition, BM-MMSC expressed high level of ALP activity and were induced to differentiate into osteoblast cells on Zn-incorporated coatings. Moreover, the antibacterial studies demonstrated that the Zn-incorporated coatings greatly inhibited the growth of both *Staphylococcus aureus* (*S. aureus*) and *Escherichia coli* (*E. coli*). The authors [26,27] established the effective antibacterial activity in vitro of the copper (Cu)-incorporated coatings against *E. coli* bacterial colonization. Thus, these coatings were not cytotoxic and were able to stimulate the expression of angiogenic genes in BMSCs and to induce bone-like apatite nucleation and growth.

The antimicrobial features of Zn and Cu correspond to current trends in the field of cationic substituted hydroxyapatite (HA) and CaP coatings for orthopedic solutions [30]. On the other hand, Zn and Cu are essential elements, which are able to promote osteoblasts growth in vitro and bone formation and regeneration in vivo [30,31]. Shi et al. [32] reported that Cu^2+^-doped hydroxyapatite (HA) microspheres had a larger specific surface area, better hydrophilicity and stronger ability to adsorb bovine serum albumin (BSA) compared with the HA microspheres. The rat calvarial osteoblasts (RCOs), grew on Cu^2+^-doped HA layer, demonstrated greater spreading than those on the HA layer.

Nevertheless, the differences between in vitro and in vivo studies are controversial in the reported bioactivity and toxicity depending on dopant doses and processing technique [31]. We have recently demonstrated in vitro dissolution, biocompatibility with bone marrow cells and bacteriostatic effect against *S. aureus* of Zn- or Cu-containing CaP coatings, deposited on Ti and Ti-40Nb substrates by MAO method [33]. However, the regularities between the physicochemical properties of the biocoatings on metallic substrates and the cell response requires further studies. Currently, the investigation of the wettability, osteogenic in vitro and in vivo properties of MAO coatings, modified with essential trace elements to improve osseointegration and bacterial adhesion resistance on the surface of metal implants is an important task for the biomedical applications.

In the previous part of the study [34] we synthesized the CaP, Zn-containing CaP (Zn-CaP) and Cu-containing CaP (Cu-CaP) coatings on Ti and Ti-40Nb alloy by the MAO method and characterized their microstructure, phase and elemental compositions, physical, chemical, and mechanical properties. This work focuses on the subsequent studies of the wettability and biological performance in vitro and in vivo of the produced biocoatings.

## 2. Materials and Methods

### 2.1. Materials and MAO Treatment

The experimental samples used in this study were cut from billets of commercial pure titanium (Grade 2, VSMPO-AVISMA Corp., Verkhnaya Salda, Russia) and Ti-40wt.%Nb alloy (Ti-40Nb, General Research Institute for Nonferrous Metals, Beijing, China) in the form of plates of 10 × 10 × 1 mm^3^ in size as we reported elsewhere [34]. In addition, the procedures of samples preparation and subsequent coatings deposition by the MAO method were described in details in [34].

### 2.2. Wettability and Surface Energy Characterization

Wettability and surface energy of the coatings were investigated using drop shape analysis system Easy Drop DSA1 (Kruss, Hamburg, Germany). To measure the contact angles on the sample surface the liquid droplet images were analyzed by a sessile drop method. 5 µL liquid droplets were used to minimize any gravitational effects. Three samples from each group were selected for contact angle measurements. Measurements were taken 5 s after deposing a droplet on the sample surface under ambient conditions (atmospheric pressure and temperature 24 °C). Both polar (water) and nonpolar (glycerol) liquids were used to calculate the free surface energy of the coatings according to the Owens–Wendt equation [35]:

(1) $$\sigma_{L}(\cos\theta +1)=2\left(\sqrt{\sigma_{S}^{P}\sigma_{L}^{P}}+\sqrt{\sigma_{S}^{D}\sigma_{L}^{D}}\right)$$

Here, $\sigma_{S}^{D}$ and $\sigma_{S}^{P}$ are the dispersive and polar components of the solid free surface energy; $\sigma_{L}^{D}$ and $\sigma_{L}^{P}$ are the dispersive and polar components of the liquid surface tension ($\sigma_{L}$), respectively.

### 2.3. Biological Studies In Vitro

To carry out biological studies in vitro the CaP, Zn-CaP, and Cu-CaP coatings were deposited on Ti and Ti-40Nb substrates in the optimal mode with an applied voltage of 200 V for 10 min. This mode was selected in the previous part of the work [34]. Each test group was represented by three samples. Before biological studies, the samples were sterilized in an autoclave FD53 (Binder GmbH, Tuttlingen, Germany) at 180 °C for 1 h. To obtain the extracts of the MAO coatings, the samples were immersed in the RPMI 1640 synthetic culture medium at 37 °C for 7 days. 2 mL medium per each sample was used according to ISO 10993-5-2009.

The culture of adult human adipose-derived multipotent mesenchymal stromal cells (hAMMSC) was used. The hAMMSC were isolated from lipoaspirates of healthy volunteers (Permission No. 7 from 9 December 2015; the Local Ethics Committee, Innovation Park, Immanuel Kant Baltic Federal University) as described in [36]. Over 98% of viable cells expressed CD73, CD90 or CD105 markers and did not express CD45, CD34, CD20 or CD14 markers (below 2%) (see Section 3.2.1) when stained by a MSC Phenotyping Kit, human (130-095-198, Miltenyi Biotec, Bergisch-Gladbach, Germany). Specific monoclonal antibodies were labeled with fluorescein isothiocyanate (FITC), allophycocyanin (APC), phycoerythrin (PE), or peridinin chlorophyll protein (PERCP). The results of staining were analyzed with MACS Quant flow cytometer (Miltenyi Biotec, Bergisch-Gladbach, Germany) and KALUZA Analysis (Beckman Coulter, Brea, CA, USA) according to the manufacturers’ instructions.

Multilineage nature of 21-day hAMMSC culture was confirmed by staining of adherent cells with alcian blue, alizarin red S, and oil red (Sigma-Aldrich, St. Louis, MO, USA) (see Section 3.2.1). Thereto, medium with reagent based on StemPro^®^ Differentiation Kit (Thermo Fisher Scientific, Waltham, MA, USA) was used [37] according to the recommendations of the International Federation for Adipose Therapeutics and Science (IFATS) and the International Society for Cellular Therapy (ISCT) [38,39].

#### 2.3.1. Cell-IQ In Vitro Visualization of Cell Morphology and Motility

Live hAMMSC culture was observed in situ using Cell-IQ^®^ v2 MLF integrated platform (CM Technologies Oy, Tampere, Finland) as described in [40]. To study the cell morphology and motility, the samples were vertically attached by clips to the wall of a sterile well of a 12-well plastic culture flat-bottom plates (Orange Scientific, Braine-l’Alleud, Belgium). The samples can be not shifted in such positions and can be not damage the forming cell layer. Then, 70 μL hAMMSC suspension (5 × 10^4^ viable cells) was added into each well. The cells were cultured and adhered to the plastic wells in a moist chamber for 120 min. The nonadherent cells were washed off with Dulbecco’s modified Eagle’s medium/nutrient mixture F-12 Ham (DMEM) (Gibco Life Technologies, Grand Island, NY, USA). Then, the wells were filled with 1.5 mL 90% DMEM supplemented with 10% fetal bovine serum (Sigma-Aldrich, St. Louis, MO, USA), 50 mg/L gentamicin (Invitrogen, Carlsbad, CA, USA), and 280 mg/L L-glutamine (Sigma-Aldrich, St. Louis, MO, USA). The samples were cultured in 5% carbon dioxide atmosphere at 100% humidity and at 37 °C for 6 days until cell monolayer formation. They did not directly contact with cells for a long time, which determined an indirect (via coating dissolution products) impact of the three-dimensional culturing on cell migration. Sample-free hAMMSC culture was used as a control group.

**Figure 1:**
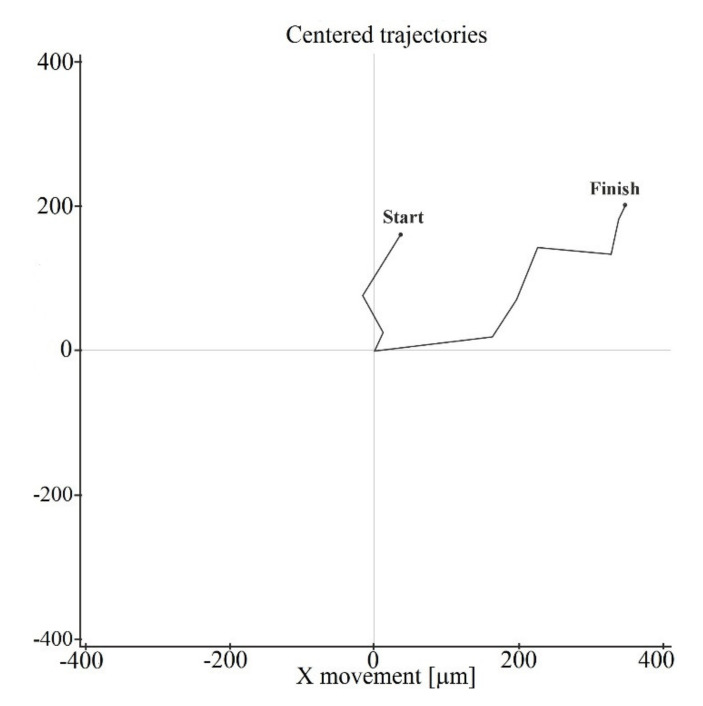
Track of some changes in hAMMSC movement in vitro.

To analyze the cell morphology and motility, the digital microphotographs were taken each 45 min for 7 days. Figure 1 shows, that the cell moved along a complex trajectory with linear sections. During manual processing of digital videos, linear distances were evaluated by displacement of the cell nucleus due to significant variation in the shape and sizes (up to 100–400 µm in length) of migrating hAMMSC. The average linear velocity of free movement (ALVFM) of cells was determined before they formed cell-cell contacts.

#### 2.3.2. Osteocalcin and Ion Concentration Estimation In Vitro

Osteogenic differentiation of hAMMSC was evaluated by osteocalcin (OC) secretion measurement in supernatants in vitro. The supernatants (intercellular fluids, conditioned media) were obtained by centrifugation of a 6-day cell culture for 10 min at 500× *g*, cultivated either in standard DMEM or in osteogenic medium StemPro^®^ Differentiation Kit (Thermo Fisher Scientific, Waltham, MA, USA). The OC concentration was estimated using Osteometer BioTech A/S N-MID Osteocalcin One Step ELISA test system (Nordicbioscience diagnostics, Herlev, Denmark).

The analysis was performed by the standard ELISA assay. Concentration of calcium ions (Ca^2+^), phosphate ions (PO_4_^3−^), and calcium salts (Ca total) in supernatants were evaluated by ion-selective electrodes with a biochemical automatic analyzer Konelab60i (Thermo Fisher Scientific, Chicago, IL, USA) according to the to the manufacturers’ protocols.

### 2.4. Antibacterial Test In Vitro

The antibacterial activity of CaP, Zn-CaP and Cu-CaP-coatings deposited both on Ti and Ti-40Nb substrates was evaluated using pathogenic strain *S. aureus* 209P (the collection of the Department of Microbiology of Siberian State Medical University, Tomsk, Russia) as described previously [33]. The extracts of the MAO coatings were obtained by the sample incubation in 0.9% NaCl solution or synthetic RPMI-1640 medium (Sigma-Aldrich, St. Louis, MO, USA) for 7 days at 37 °C (2 mL medium per sample was used according to ISO 10993-5-2009). To prepare a bacterial suspension, *S. aureus* (500 microbial bodies) was placed in 15 mL plastic tubes with the 7-day sample extracts or sample-free media (0.9% NaCl solution, RPMI-1640 medium) at the proportion of 1:1 (0.5 mL: 0.5 mL) and was incubated for 2 h at 37 °C. Then, 0.2 mL bacterial suspension was placed into nutrient agar medium in 90 mm plastic Petri dishes and was cultured for 24 h at 37 °C, and 100% humidity. Three Petri dishes for each group were used. The method of computer morphometry was used (software measurements by Image J 1.38, National Institutes of Health, Bethesda, MD, USA) to measure the areas of *S. aureus* growth because of the numerous sites of microbial cell crowding.

### 2.5. Ectopic Osteogenesis Test in Mice

In vivo study was carried out on 27 Balb/c male mice in compliance with the principles for the humane treatment of laboratory animals specified in [41]. The animal experiments were approved by the Local Ethics Committee of Siberian State Medical University (Permission No. 948 from 9 February 2009). In this study, 18 animals were used for the implantation and 9 animals served as syngeneic bone marrow donors. The samples were incubated in vitro with bone marrow column from mouse femurs for 45 min and implanted under etherisation into the lateral subcutaneous pocket of the animal venter as described in [42]. Bone marrow served as a well-known source of multipotent mesenchymal stem cells (MMSC) to promote ectopic bone formation [43].

The bone tissue with or without bone marrow, grown on de novo after 45-day implantation, was examined in histological sections of grown tissue lamellae as a positive outcome of the ectopic osteogenesis test on the sample surface. To determine the incidence of new bone formation on the sample surface, the thin (5–10 μm) cross-sections of the middle part of the lamellae stained with hematoxylin-eosin were estimated using optical microscope Axioscop40 (Carl Zeiss, Oberkochen Germany), equipped with digital camera Canon Power Shot A 630.

### 2.6. Statistical Analysis

Statistical analysis was carried out using the Statistica 13.3 software. Data of the wettability and biological studies are presented as mean (X) ± standard deviation (SD) or as median (Me), 25% (Q1) and 75% (Q3) quartiles. The normality of distribution was defined by Kolmogorov-Smirnov test. Because of non-normal distribution and non-parametric Mann–Whitney U-test were used to evaluate the significant differences between the groups. Statistically significant difference was considered at value of *p* < 0.05.

## 3. Results and Discussion

### 3.1. Wettability and Free Surface Energy

It is well known that an increase in the surface energy can lead to increasing surface wettability [44]. Higher surface energy and lower contact angle supports cell adhesion, proliferation, and differentiation to a greater extent than biomaterials with a low surface energy [45]. Park et al. [46] noted that surface hydrophilicity influences the adsorption of cell adhesion proteins (e.g., fibronectin, vitronectin, collagen, and laminin of fibrin) on the implant surface, and thus enhances the adhesion and spreading of osteoblast precursors on the implant surfaces. The authors [47,48] agreed that highly wettable surfaces also enhance early bone healing process in the cell/biomaterial interface by increasing adsorption of these extracellular matrix proteins, and improving subsequent cell behavior on these surfaces.

**Figure 2:**
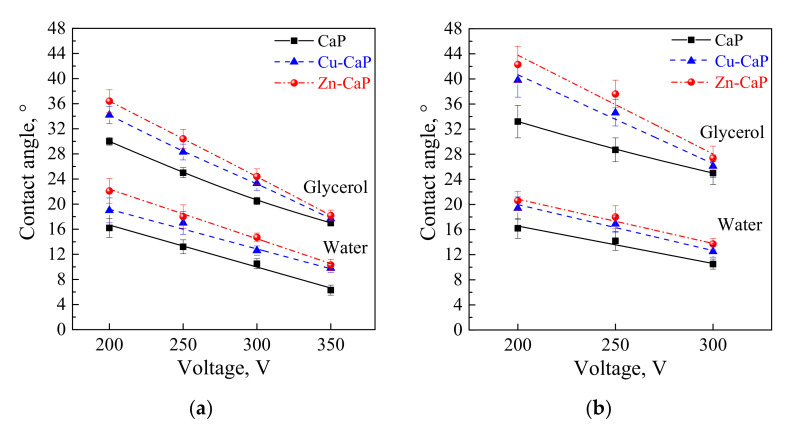
Graphs of the contact angles with water and glycerol of MAO-coatings on Ti (**a**) and Ti-40Nb (**b**) against the applied voltage.

Table 1 represents data of the water and glycerol contact angles on the surface of the CaP, Zn-CaP, and Cu-CaP coatings on both substrates (Ti and Ti-40Nb) and data of the free surface energy. The low contact angles, that is below 44°, indicate high hydrophilicity for all coating types. An increase in the MAO voltage leads to a linear decrease of the water contact angles on all the coating types from 23° to 6° (Figure 2). The same negatively related regularities are observed for the glycerol contact angles on the Ti and Ti-40Nb substrates from 37° to 17° and from 43° to 26°, respectively. The previous article [34] showed that an increase in the applied voltage leads to a linear increase in the coating surface roughness and surface porosity (Table 1) which can be accompanied by a decrease in the contact angles with liquids. Furthermore, a growth in the size of structural elements and formation of monetite plate-like crystals with length up to 15 µm on the coating surface [34] promotes an increase of the specific area and, consequently, of the coating wettability. Figure 2 demonstrates that both substrates, modified with Zn and Cu coatings, are characterized by higher values of the water and glycerol contact angles compared with the CaP coatings, deposited at the same applied voltages. This behavior is possible due to the incorporation of the Zn^2+^ and Cu^2+^ metal ions into the CaP coatings which prevents wetting.

The dispersive and polar components of surface energy as well as full surface energy for all the coating types were calculated according to Equation (1). The dispersive component of free surface energy results from molecular interaction due to the London forces which are the part of the van der Waals forces [49]. These forces represent the weak intermolecular forces, arising from quantum-induced instantaneous polarization multipoles in molecules. The polar component of surface energy comprises all other interactions due to non-London forces. Polar molecules interact through dipole/dipole intermolecular forces as well as hydrogen or phosphate bonds [49].

Table 1 demonstrates that the polar component significantly prevails over the dispersive component of the surface energy. It can indicate strong polar chemical bonds in the coatings, e.g., phosphate and hydroxyl bonds. These data are confirmed by the previous results of infrared spectroscopy and X-ray diffraction [34]. Intensive adsorption bands from P–O and OH-bonds were observed in the infrared spectra. In addition, the amorphous CaP phase and crystalline CaHPO_4_ and Ca(H_2_PO_4_)_2_·H_2_O phases were observed in XRD spectra [34]. It is known that hydrophilic surfaces are characterized by a high polar component of the surface energy. Harnett et al. [50] showed that cell adhesion and proliferation mainly depend on the polar component and significantly increase with an increase in the polar component of the surface energy.

An increase in the MAO voltage from 200 to 350 V leads to a decrease in the polar component and an increase in the dispersive component for all the coating types (Table 1). This is due to an increase in the coating roughness which results in an increase in the non-polar van der Waals forces. However, polar bonds on the coating surface still predominant. An increase in the applied voltage leads to a decrease in free surface energy from 76 to 72 mJ/m^2^ for the coatings on Ti and from 81 to 74 mJ/m^2^ for the coatings on Ti-40Nb alloy. A slight decrease in the surface energy, caused by an increase in the surface roughness, leads to weakening of the attractive forces between the liquid and solid molecules which create energy on the coating surface. It should be noted that the surface energy values of 72–81 mJ/m^2^ indicates good wetting of all coating types on the both substrates. These values exceed the surface tension values of the most liquids (e.g., surface tension of water is 72.8 mJ/m^2^, surface tension of glycerol is 64 mJ/m^2^, and surface tension of diiodomethane is 50.8 mJ/m^2^ [35]). According to [51], hydrophilic surfaces promote adherence and proliferation of fibroblasts, which apparently is one of mechanisms of preferential growth of connective tissue on the MAO coatings.

### 3.2. Biological Performance In Vitro

#### 3.2.1. Multilineage Nature of hAMMSC Culture

**Figure 3:**
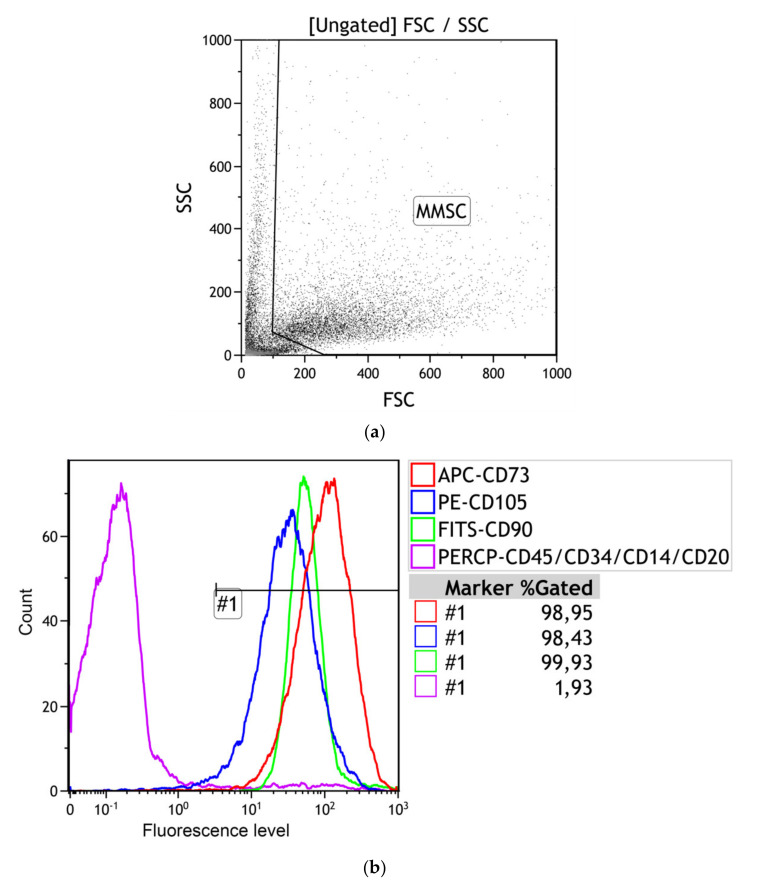
A strategy for gating live hAMMSCs. (**a**) MMSC identification based on forward scatter (FSC) vs. side scatter (SSC); (**b**) CD73+, CD90+, and CD105 + vs. CD14+CD20+CD34+CD45+.

In standard nutrition medium without differentiation supplements, 98–99% of human adipose-derived adherent cells expressed CD73, CD90, and CD105 markers and did not display CD45, CD34, CD20, and CD14 antigens (less 2%) (Table 2, Figure 3).

**Figure 4:**
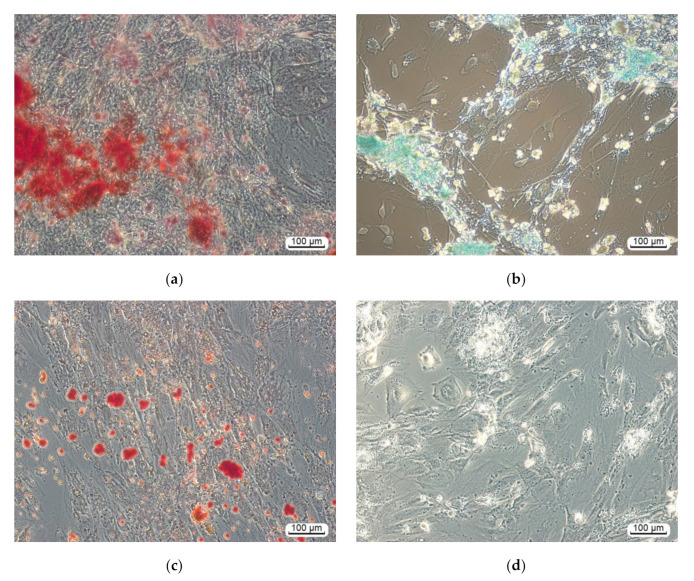
hAMMSCs cultured for 21 days in either (**a**–**c**) StemPro^®^ differentiation or (**d**) standard media: (**a**) osteogenic medium, alizarin-red-stained areas of mineralized regions of the intercellular substance; (**b**) chondrogenic medium, alcian-blue-stained glycoproteins; (**c**) adipogenic medium, oil-red-stained neutral triglycerides, and lipids; (**d**) some adipose-derived cells cultivated in the standard medium contain light fatty inclusions (unstained).

Hereafter, human adipose-derived fibroblast-like CD73CD90CD105^+^ cells adhered to plastic wells in a StemPro^®^ Differentiation Kit (Thermo Fisher Scientific, USA) for 21 days were positively stained with alizarin red (osteoblasts), alcian blue (chondrocytes), and oil red (adipocytes) (Figure 4a–c) as compared with unstained cells in standard medium (Figure 4d). Overall, these findings showed that the cells corresponded to the minimal morphological criteria of the multipotent MSCs according to the recommendations of the IFATS and the ISCT [38,39]. The results obtained corresponds to our previous data [37].

#### 3.2.2. hAMMSC Morphology and Motility

**Figure 5:**
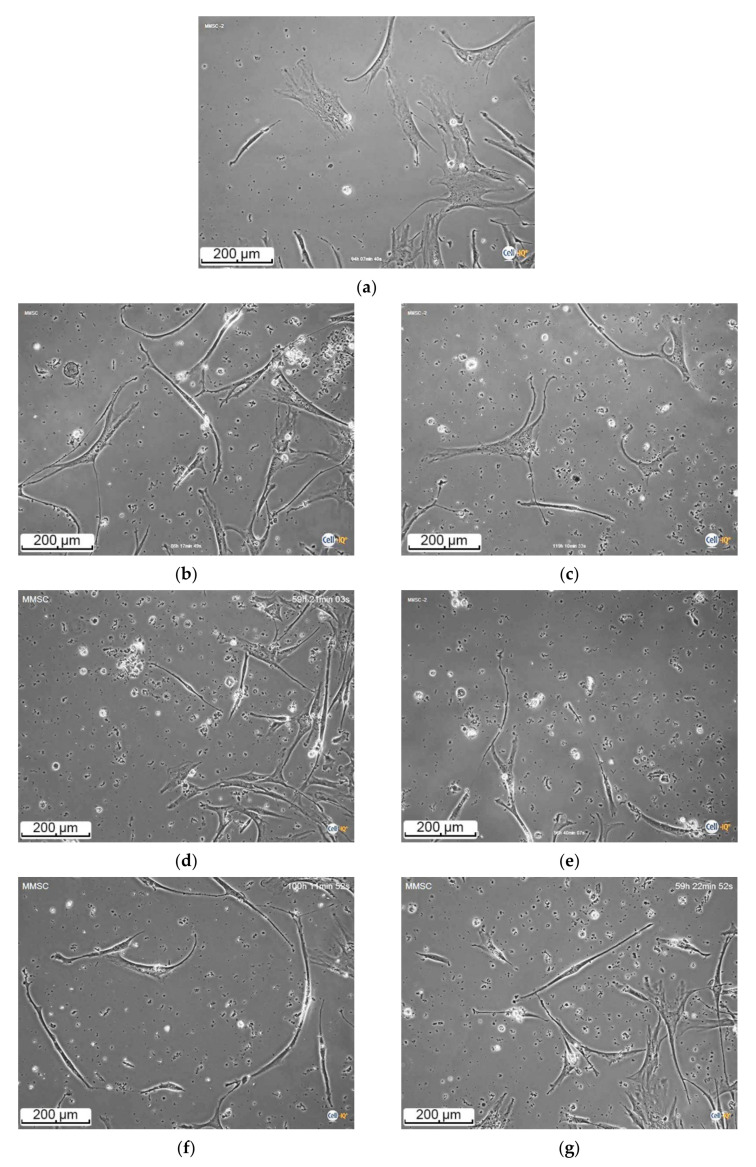
Images of Cell-IQ phase-contrast real-time microscopy of unstained hAMMSC co-cultured with the samples for 6 days in vitro: (**a**) sample-free DMEM; (**b**) CaP coating on Ti; (**c**) CaP coating on Ti-40Nb; (**d**) Zn-CaP coating on Ti; (**e**) Zn-CaP coating on Ti-40Nb; (**f**) Cu-CaP coating on Ti; (**g**) Cu-CaP coating on Ti-40Nb.

Cell-IQ continuous real-time dark-field microscopy showed the fibroblast-like and spindle-shaped adherent hAMMSC in sample-free culture medium (control group) (Figure 5a). Observed hAMMSC had linear sizes of 100–400 µm and ALVFM was ~35 µm/h (Table 3). In cases of indirect contacts with all types of the MAO coatings (via the biodegradation products), migrating cells had a similar morphology (Figure 5b–g). The hAMMSC adhered to the coatings, increased their cell mass, and migrated actively for 6 days until the monolayer was formed.

Physical properties of the coating surface significantly influence the stromal cell behavior [37,52,53,54]. All MAO coatings, formed on both substrates, had similar topography properties (Table 1 and Table 3). However, only the Zn-CaP coating on Ti-40Nb substrate increased the ALVFM of hAMMSC by 24% (*p* < 0.05) compared with the CaP coating (Table 3). It is to note that the samples were placed vertically at one of the edges of culture plates thereby preventing their direct contact with cells. In this case, the MAO coatings influenced the hAMMSC behavior only through their biodegradation products in vitro. The most positive effect on the increased cell ALVFM, compared with other coating types, was demonstrated by the Zn-CaP coatings on the Ti-40Nb substrate. According to [33] this is due to the release of Zn^2+^ ions, which have attractive properties for cells, into the culture medium. The review [30] also noted that doping of HA with Zn^2+^ increased the osteoblast cell viability, adhesion, spreading, proliferation, differentiation, and stimulated osteogenic activity, bone in-growth and healing. Currently, Zn-dependent metalloenzyme action is widely studied at the cellular and supramolecular levels. Thus, Yamaguchi et al. [55] showed in vitro that 10^−5–^10^−4^ M zinc sulfate in culture medium led to significant increase in Runx2, osteoprotegerin and regucalcin mRNA expressions in osteoblastic MC3T3-E1 cells.

#### 3.2.3. Osteocalcin and Ion Concentrations in Supernatants

An increasing number of researchers recommends advanced biomimicry testing of the biomineralization capability of bioactive materials, using cell cultures media supplemented with 10% serum at 37 °C, in a humid atmosphere with 5% partial pressure of CO_2_ (as found in living tissues) instead of simulated body fluid (SBF) assay under normal atmospheric conditions [30]. In [56,57], osteoblasts, differentiated from MMSC, increased deposition of calcium salts from intercellular ions by ALP production. Cells are capable to dissolve natural and artificial CaP materials through their enzymes for example acid phosphatase. Advanced in vitro biomimetic conditions, required for the investigation of correct biomineralization/dissolution of CaP materials, relate to a presence of MMSC/osteoblasts and some other cells (osteoclasts, monocytes, etc.) to dissolve and/or precipitate calcium phosphates. The works [56,57] described these processes of CaP precipitation and deposition (calcification) on both osteoblasts differentiated from MMSC and artificial surfaces of the samples.

ALP and OC are considered as the true molecular markers of MMSC differentiation into secretory osteoblasts [58]. Previously [59], we showed in vitro that unlike the Zn-CaP coatings both on Ti and Ti-40Nb substrates, the Cu-CaP coatings had a stimulating effect on ALP activity in supernatants of prenatal MMSC of the human lung. It was an unconventional result, because ALP is a Zn-dependent enzyme with three closely spaced metal ions (two Zn ions and one Mg ion) at the active center [60], and Zn can stabilize the ALP and increase its half-life [61].

This in vitro study shows that special differentiation medium with osteogenic supplements caused twice an increase of OC concentration in supernatants of hAMMSC, cultivated for 6 days compared with DMEM (control group) (Table 3). All tested samples demonstrated a tendency to accelerate OC secretion by 19–72% compared with the control group. In this case, the Cu-CaP coatings on the both substrates showed maximal OC concentration in supernatants. Their values were 72% higher for the Cu-CaP coating on pure Ti and 47% higher for the Cu-CaP coating on the Ti-40Nb alloy than that of the control group. The Cu-CaP coatings can positively affect osteogenesis by differentiation of bone marrow stem cells, enhanced angiogenesis and increased collagen formation in the extracellular matrix (ECM), and the precipitation and subsequent mineralization of calcium phosphates onto the ECM, caused by OC and ALP [31]. Thus, in the case of the osteogenic Cu-CaP coatings, ALP and OC can be molecular pathways for inducing protein adsorption, as well as nucleation and growth of bone apatite at implantation site, as described in [27,32].

Further, despite a good solubility in vitro of the MAO coatings [57,59,62], the concentrations of calcium salts (Ca total) decreased statistically by 8–11% (*p* < 0.05) in supernatants of hAMMSC, co-cultured with all the tested coatings except for the Zn-CaP coating on the Ti-40Nb substrate (Table 3). The same results were received earlier for in vitro of prenatal MMSC of the human lung, contacted with the CaP-coatings on pure Ti samples [57]. In addition, we observed sporadic decrease of Ca^2+^ and PO_4_^3−^ concentrations that indicate the precipitation of amorphous calcium phosphates and deposition (calcification) on cells and artificial surfaces.

Thus, low Cu and Zn contents (~0.4 at.%) [33,34] in the CaP coatings on both Ti and Ti-40Nb substrates promoted in vitro hAMMSC ability to differentiate into osteoblasts. Moreover, the Zn- CaP coating stimulated in vitro the hAMMSC motility on the implant-cells interface promoting osteoconduction mechanisms. A good inflow of mesenchymal cells to the fracture site is key to the formation of high-grade bone [63,64].

### 3.3. Antibacterial Efficacy In Vitro

In 2013, on the International Consensus Meeting on Periprosthetic Joint Infection (Philadelphia, PA, USA) it was concluded that surgical site infections and periprosthetic joint infection, with or without its serious implications, continued to pose a challenge to the orthopedic community [65]. Furthermore, critical point of implant osteointegration/failure is an interface between artificial surface and bone cells and tissue. Bacterial biofilm disturbs close bone/implant interface. In turn, implant mechanical instability promotes infectious complications and leads to implant failure [66].

**Figure 6:**
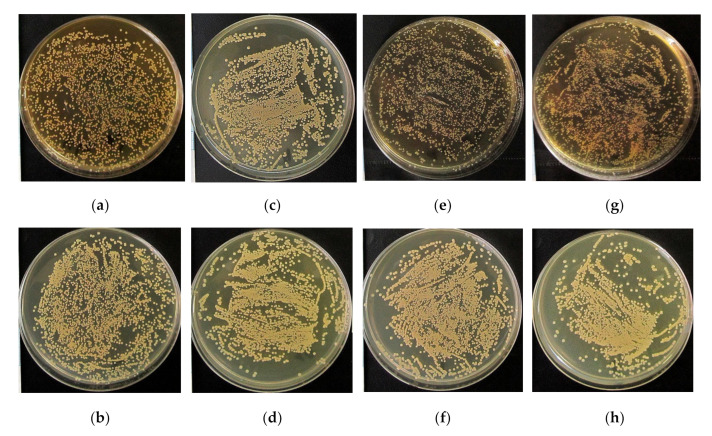
Photographs of a 24 h *S. aureus* culture in agar medium after 2 h pre-incubation with: (**a**) 0.9% NaCl (control 1); (**b**) RPMI-1640 (control 2); (**c**) extract of CaP coating on Ti; (**d**) extract of CaP coating on Ti-40Nb; (**e**) extract of Zn-CaP coating on Ti; (**f**) extract of Zn-CaP coating on Ti-40Nb; (**g**) extract of Cu-CaP coating on Ti; (**h**) extract of Cu-CaP coating on Ti-40Nb.

In this context, the antibacterial activity of the CaP, Zn-CaP and Cu-CaP coatings both on Ti and Ti-40Nb substrates against *S. aureus* was studied in vitro. Figure 6 shows the photographs of 24 h *S. aureus* culture in agar medium after a 2 h preliminary incubation with 7-day extracts of the MAO coatings or with nutrient media (0.9% NaCl solution, RPMI-1640), used as control groups. A diameter of each microbial colony forming unit (CFU) reached 0.5–1.0 mm. The colonies had the correct S-type shape and were colored with golden pigment. It was impossible to detect the number of individual CFUs because of the numerous clusters of microbial cells. Therefore, the relative area of microbial colonies per total area of Petri dish was calculated (Table 4).

After the 24 h incubation period and exposure to RPMI-1640 medium and extracts of CaP coating without modifications on both substrates, a significant increase in the area of *S. aureus* colonies in the agar medium was observed (Figure 6a–d). The relative area of *S. aureus* colonies increased by 14–15% for RPMI-1640 medium and by 12–17% for CaP coating extract compared with the 0.9% NaCl solution (control 1) (Table 4). The result of RPMI-1640 medium was predictable since it contains nutrients such as glucose, amino acids, and vitamins [33]. It is worth noting, that the obtained values of the relative area of *S. aureus* colonies per Petri dish for all the studied groups did not exceed 45%. The extracts of the Zn-CaP and Cu-CaP coatings on both substrates also stimulated a small increase in the area of the *S. aureus* colonies, which did not exceed 10%, compared with the control group 1. The Zn-CaP coating on Ti was the only exception, where the relative area of *S. aureus* colonies was 25–27% (Figure 6e–h). However, in cases when the RPMI-1640 nutrient medium was used as a control group (control 2), the extracts of Zn-CaP and Cu-CaP coatings on both substrates showed an antibacterial effect, especially the Zn-CaP coating on Ti. The relative area of *S. aureus* colonies decreased by 3–6% for the extract of Cu-CaP coating on Ti, by 7–10% for the extracts of Zn-CaP and Cu-CaP coatings on Ti-40Nb, and by 15–18% for the extract of Zn- CaP coating on Ti compared with RPMI-1640 medium (control 2) (Table 4). Thus, the extracts of Zn- or Cu-containing MAO coatings on both substrates may be arranged by their antibacterial efficacy as follows: Zn-CaP/Ti > Cu-CaP/TiNb, Zn-CaP/TiNb > Cu-CaP/Ti (Table 4). The obtained data correspond to the results of other authors [20,26] and our previous results for MAO coatings [33].

### 3.4. Ectopic Osteogenesis in Mice

Nepola et al. [66] considered the biocompatibility and functional activity of implants for osteosynthesis depending on the processes occurring in the bone/implant interface. The desired features of the MAO coatings to induce MMSC osteogenic differentiation was achieved in vitro [37]. In the absence of in vivo pieces of evidence of enhancing osteogenesis, caused by such materials, there is a risk of bone healing failure in surgical practice. Therefore, animal studies could provide accurate results.

Reparative bone remodeling occurs via the attraction of mesenchymal cells and their subsequent differentiation into osteoblasts and via the activation of osteoblasts, located in the damage site, and via osteoconduction processes [67]. BM-MMSC and AMMSC are commonly used as precursors of stromal stem cells in skeletal tissue engineering in vitro and in vivo [68]. In this case, BM-MMSC pool is more prone to osteogenic differentiation than AMMSC and shows superior ectopic bone formation without additional growth factors [69]. Experimentally induced ectopic bone formation with subcutaneous transplantation is one of well-studied valid models crucial to understand in vivo osteogenic differentiation of MMSC independently from an osseous microenvironment [70] and to study the osteoinduction potential [71].

**Figure 7:**
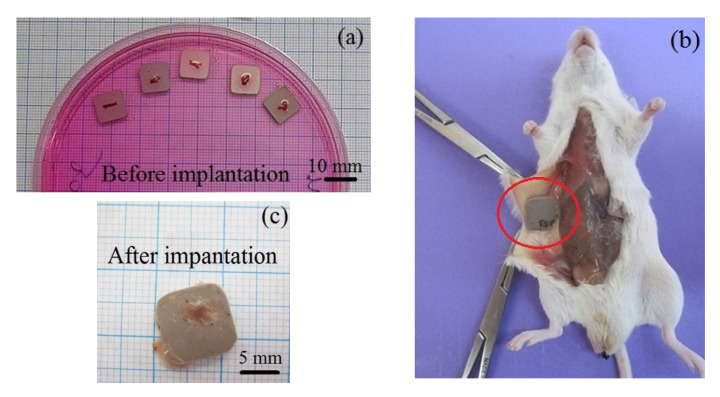
Photographs of the samples with in vitro applied bone marrow before implantation (**a**) and after 45-day subcutaneous implantation in vivo (**b**,**c**).

To define the osteogenic properties of the MAO coatings, we carried out a test of ectopic bone formation in situ. In this test, the artificial samples with in vitro applied bone marrow were implanted under mouse skin without injection of additional growth factors (Figure 7) [62]. After 45 days, no signs of the inflammation and tissue sensation were observed in the sites of the subcutaneous implantation of all the samples. All samples were surrounded by a thin (<50 µm) stromal connective tissue capsule which easily removed (Figure 7). The incidence of in situ formation of tissue lamellae from the bone marrow supplied in vitro was 100% in all the studied groups (Table 5).

**Figure 8:**
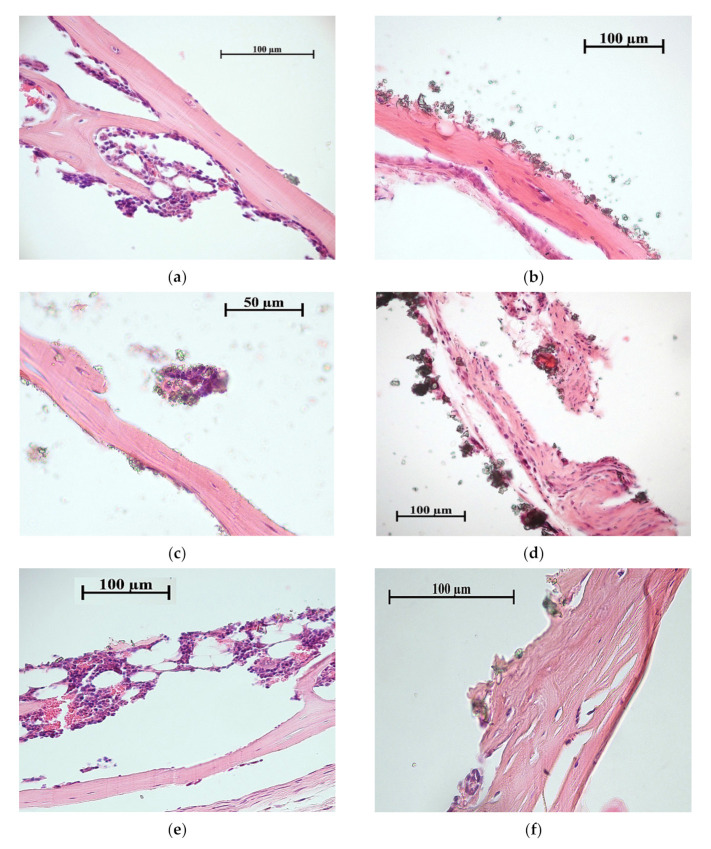
Histological composition of bone lamellae grown on the Ti substrate: (**a**,**b**) CaP coating; (**c**,**d**) Zn-CaP coating; (**e**,**f**) Cu-CaP coating. Bone with marrow (**a**,**e**), bone lamellae (**b**,**c**,**f**), and the sites of connective tissue ossification (**d**) are shown. Hematoxylin—eosin staining.

**Figure 9:**
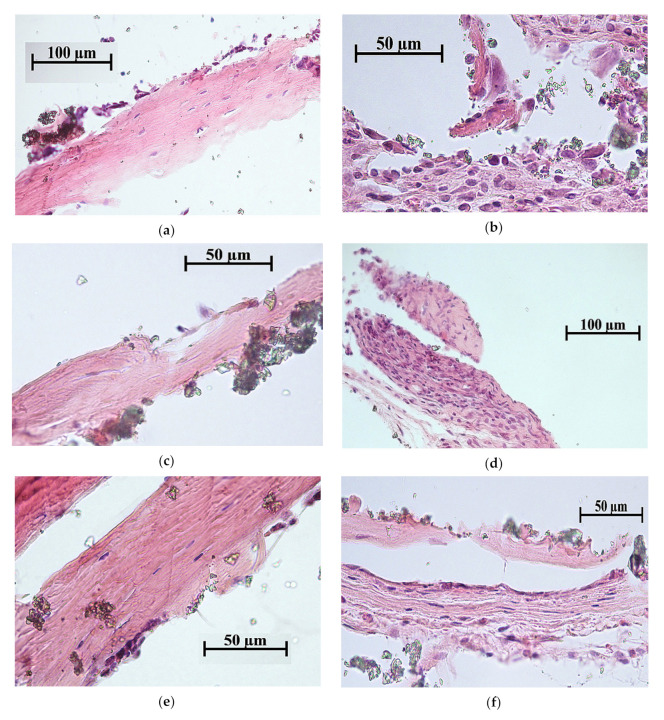
Histological composition of bone lamellae, grown on the Ti-40Nb substrate: (**a**,**b**) CaP coating; (**c**,**d**) Zn-CaP coating; (**e**,**f**) Cu-CaP coating. Bone lamellae (**a**,**c**,**e**) and the sites of connective tissue ossification (**b**,**d**,**f**) are shown. Hematoxylin – eosin staining.

The bone with (33% incidence) or without bone marrow (67% incidence) was observed in 100% tissue lamellae for all coatings on Ti substrate (Figure 8, Table 5). In the case of CaP coating without additives, thin (<20 μm) layers of connective tissue covered some bone lamellae (Figure 8b). The Zn-CaP and Cu-CaP coatings decreased the incidence of bone formation in tissue lamellae down to 67% (Table 5). Bone lamellae (Figure 8c) and connective tissue ossification sites (Figure 8d) were found on the surface of Zn-CaP coatings. At the same time, the bone with marrow (Figure 8e) and bone lamella (Figure 8f) were formed on the surface of Cu-CaP coatings. Bone marrow metaplasia into vascularized loose irregular connective tissue on the coating surface was classified as a failure of implantation. In the case of all coatings on the Ti-40Nb alloy, bone lamellae and connective tissue ossification sites were formed with 67% of incidences (Figure 9, Table 5). For other 33% of incidences, adipose, muscle, and/or connective tissue were observed.

Thus, only the CaP coating without additives on Ti substrate showed 100% new bone formation in situ (Figure 8a,b; Table 5) that agree with our previous data [62]. The bone engineering via stage of cartilage (endochondral ossification) is required for remodeling of hematopoietic tissue from the hematopoietic stem cells. Another way is the formation of bone without bone marrow by stromal stem cells, bypassing the stage of cartilage, by ossification of connective tissue [72]. In this study, both ways of the ectopic ossification occurred (Figure 8 and Figure 9; Table 5).

Slightly impaired ectopic osteogenesis (down to 67% incidence of bone formation) was detected on the surface of Zn- and Cu-doped CaP coatings on both metal substrates (Table 5). At least, the results do not differ dramatically from 67–100% ectopic bone remodeling determined previously for MAO CaP coating [62].

Local (tissue microenvironment), distant (regulatory systems) factors and mechanical forces exerted on an implant site should be considered for osteogenesis outcome [62,70,73]. Kawamura et al. [74] showed in vivo that optimum Zn content of ~0.3 wt.% in the CaP ceramics promoted bone formation while ~0.6 wt.% Zn content increased bone resorption.

## 4. Conclusions

The studies of the wettability and biological properties of the CaP and modified Zn-CaP or Cu-CaP coatings, prepared by MAO on pure Ti and Ti-40Nb alloy allows to draw the following conclusions.

1. It was found that highly wettable CaP, Zn-CaP and Cu-CaP coatings on both pure Ti and Ti-40Nb alloy had low contact angles with water and glycerol, which is below 45°. An increase of the applied voltage led to an increase of the coating roughness and porosity, thereby reducing the contact angles to 6° with water and to 17° with glycerol.

2. The free surface energy of the MAO coatings of 73 mJ/m^2^ for the Ti substrate and of 78 mJ/m^2^ for the Ti-40Nb substrate were determined. The main component of the surface energy was polar component indicating strong polar chemical bonds of PO_4_^3−^ and OH^−^ -groups in the coatings.

3. Low Cu and Zn contents (~0.4 at.%) in the MAO coatings both on Ti and Ti-40Nb substrates promoted in vitro hAMMSC ability to differentiate into osteoblasts. Moreover, the Zn-CaP coating on Ti-40Nb alloy stimulated in vitro hAMMSC motility as a possible mechanism of osteoconduction on the implant-cells interface.

4. The Zn- or Cu-containing MAO coatings showed antibacterial efficacy against *S. aureus* and can be arranged as follows: Zn-CaP/Ti > Cu-CaP/TiNb, Zn-CaP/TiNb > Cu-CaP/Ti.

5. Alone the CaP coating on Ti substrate showed 100% ectopic bone formation in vivo. The Zn-CaP and Cu-CaP coatings on the both substrates slightly decreased the incidence of ectopic osteogenesis down to 67%.

6. The Zn- and Cu-containing CaP coatings and the CaP coatings without additives on pure Ti and Ti-40Nb alloy are promising biomaterials and can be used for advanced medical implants for osteosynthesis complicated by osteoporosis and periprosthetic infection.

## Figures and Tables

**Table 1:** Contact angles and free surface energy of the micro-arc oxidation (MAO) coatings deposited at the different applied voltages on Ti and Ti-40Nb, X ± SD.

Type of the Coating	Voltage, V	Coating Roughness [34], µm	Coating Porosity [34], %	Water Contact Angle, °		Glycerol Contact Angle, °		Polar Component, mJ/m^2^	Dispersive Component, mJ/m^2^	Free Surface Energy, mJ/m^2^
Ti substrate										
CaP	200	2.9 ± 0.5	16.2 ± 0.8	16.2 ± 1.5	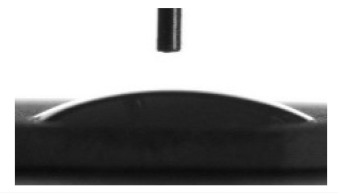	30.0 ± 0.6	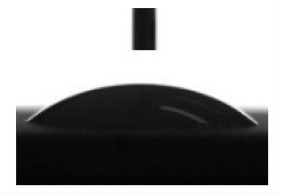	67.3 ± 0.4	7.3 ± 0.2	74.6 ± 0.5
Zn-CaP		3.2 ± 0.2	17.7 ± 0.6	22.1 ± 1.9		36.4 ± 1.8		69.9 ± 0.4	5.6 ± 0.1	75.5 ± 0.5
Cu-CaP		3.0 ± 0.2	17.8 ± 0.2	19.0 ± 1.9		34.2 ± 1.4		69.6 ± 0.8	5.2 ± 0.2	74.4 ± 1.0
CaP	250	4.0 ± 0.4	19.1 ± 0.7	13.2 ± 1.1	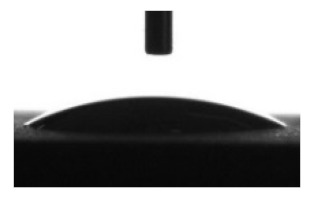	25.0 ± 0.8	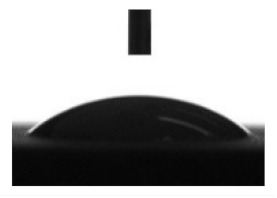	63.2 ± 0.4	10.9 ± 0.3	74.1 ± 0.8
Zn-CaP		4.5 ± 0.2	21.2 ± 0.6	18.0 ± 1.8		30.4 ± 1.5		64.6 ± 0.5	8.5 ± 0.2	73.1 ± 0.7
Cu-CaP		4.4 ± 0.4	21.6 ± 0.4	17.0 ± 1.8		28.3 ± 1.3		67.2 ± 0.7	7.3 ± 0.2	74.5 ± 0.9
CaP	300	5.0 ± 0.5	21.4 ± 0.9	10.5 ± 0.8	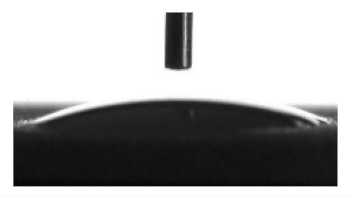	20.5 ± 0.6	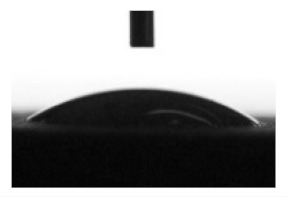	61.1 ± 1.0	11.3 ± 0.6	73.4 ± 1.6
Zn-CaP		5.9 ± 0.3	24.4 ± 0.7	14.7 ± 0.7		24.4 ± 1.2		61.1 ± 0.4	11.3 ± 0.3	72.3 ± 0.7
Cu-CaP		5.9 ± 0.2	24.1 ± 0.2	12.6 ± 0.7		23.3 ± 1.1		64.3 ± 0.3	8.6 ± 0.1	72.9 ± 0.4
CaP	350	6.3 ± 0.4	24.1 ± 1.2	6.3 ± 0.8	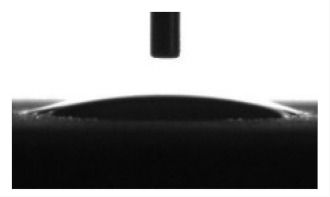	17.0 ± 0.5	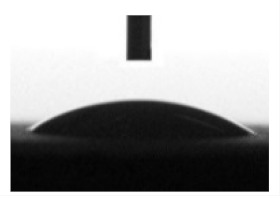	57.9 ± 0.5	12.5 ± 0.3	72.7 ± 0.6
Zn-CaP		7.2 ± 0.6	25.0 ± 1.8	10.3 ± 0.9		18.2 ± 0.8		59.9 ± 0.6	12.7 ± 0.4	72.6 ± 0.6
Cu-CaP		7.2 ± 0.4	25.2 ± 0.4	9.8 ± 0.7		17.7 ± 0.7		60.6 ± 0.5	12.3 ± 0.3	72.9 ± 0.7
**Ti-40Nb substrate**										
CaP	200	3.2 ± 0.3	15.7 ± 0.5	16.2 ± 1.6	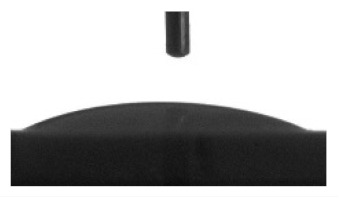	33.2 ± 2.6	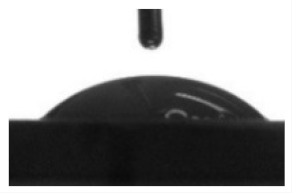	69.5 ± 0.4	6.5 ± 0.1	76.0 ± 0.5
Zn-CaP		3.3 ± 0.5	16.7 ± 0.5	20.6 ± 1.5		42.3 ± 2.9		75.3 ± 0.7	3.3 ± 0.1	78.6 ± 0.9
Cu-CaP		3.3 ± 0.4	16.7 ± 0.5	19.4 ± 1.8		39.8 ± 2.7		79.0 ± 1.0	2.4 ± 0.2	81.4 ± 1.2
CaP	250	4.7 ± 0.3	17.6 ± 0.6	14.2 ± 1.3	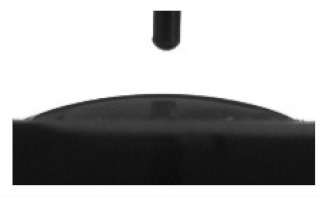	28.7 ± 1.9	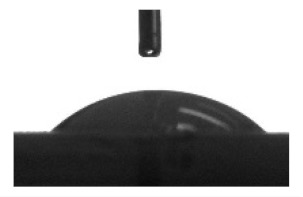	66.1 ± 0.4	8.5 ± 0.2	74.6 ± 0.6
Zn-CaP		4.8 ± 0.2	19.9 ± 0.8	18.0 ± 1.8		37.6 ± 2.2		70.6 ± 0.5	5.8 ± 0.2	76.4 ± 0.7
Cu-CaP		4.9 ± 0.4	19.6 ± 0.6	16.9 ± 1.3		34.6 ± 2.1		75.0 ± 0.4	3.9 ± 0.1	78.9 ± 0.5
CaP	300	6.0 ± 0.2	21.0 ± 0.6	10.5 ± 0.8	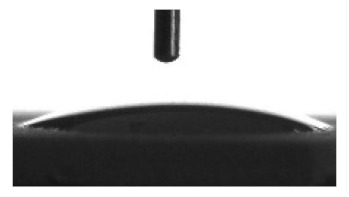	25.0 ± 1.8	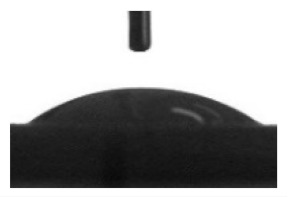	64.2 ± 0.3	10.0 ± 0.1	74.2 ± 0.5
Zn-CaP		6.9 ± 0.5	22.9 ± 1.1	13.7 ± 0.9		27.4 ± 1.9		63.1 ± 0.3	10.1 ± 0.2	74.0 ± 0.4
Cu-CaP		7.1 ± 0.2	23.0 ± 0.9	12.5 ± 0.9		26.1 ± 1.8		64.2 ± 0.3	9.9 ± 0.1	74.1 ± 0.5

**Table 2:** Immunophenotype of hAMMSCs in standard nutrition medium, Me (Q1-Q3).

Stromal Cell Markers, %			Hematopoietic Cell Markers, %
CD73	CD90	CD105	[CD45, 34, 20, 14]
98.46 (98.36–98.95)	98.57 (98.09–99.93)	98.91 (98.43–99.05)	1.34 (1.26–1.93)

Note: each measurement was done in triplicate.

**Table 3:** Cell motility, osteocalcin and inorganic ion concentrations in the supernatants of hAMMSC co-cultured for 6 days with the MAO coatings, Me (Q1–Q3), X ± SD.

No.	Groups, *n* = 3	Coating Roughness [34], μm	Coating Mass, mg	Coating Thickness [34], µm	ALVFM of Cells, µm/h	Osteocalcin Concentration, ng/mL	[Ca^2+^], mM	[Ca] Total, mM	[PO_4_^3−^], mM
1.	Cells in sample-free DMEM (control), *n* = 6	-	-	-	35 ± 11 *n*_1_ = 21	1.64 (1.44–1.78)	0.84 (0.83–0.85)	2.43 (2.41–2.53)	1.31 (1.28–1.41)
2.	Cells in sample-free osteogenic medium	-	-	-	-	3.22 * (2.82–5.48) *p*_1_ < 0.05	-	-	-
**Ti substrate**									
3.	CaP coating	2.9 (2.4–3.3)	13.5 (13.0–21.2)	48.3 (45.6–54.5)	33 ± 17*n*_1_ = 15	2.13 (2.07–2.47)	0.81 * (0.80–0.82) *p*_1_ < 0.05	2.24 * (1.91–2.31) *p*_1_ < 0.05	1.20 (1.14–1.30)
4.	Zn-CaP coating	3.2 (3.0–3.4)	14.9 (11.0–18.3)	50.2 (47.5–58.2)	42 ± 22 *n*_1_ = 10	2.13 (1.72–2.93)	0.82 (0.82–0.83)	2.17 * (2.01–2.18) *p*_1_ < 0.05	1.19 * (0.74–1.23) *p*_1_ < 0.05
5.	Cu-CaP coating	3.0 (2.8–3.2)	14.3 (14.0–19.7)	54.2 (50.2–60.5)	32 ± 19 *n*_1_ = 36	2.82 * (2.59–3.79) *p*_1_ < 0.05	0.83 (0.79–0.83)	2.24 * (2.22–2.30) *p*_1_ < 0.05	1.29 (1.18–1.30)
**Ti-40Nb substrate**									
6.	CaP coating	3.2 (2.9–3.5)	14.7 (13.8–18.4)	56.5 (51.2–60.2)	33 ± 10 *n*_1_ = 32	1.95 (1.72–2.41)	0.84 (0.82–0.86)	2.21 * (2.21–2.22) *p*_1_ < 0.05	1.27 * (1.18–1.30) *p*_1_ < 0.05
7.	Zn-CaP coating	3.3 (2.8–3.8)	15.0 (15.0–15.1)	58.3 (54.5–61.5)	41 ± 11 * n_1_ = 37 *p*_6_ < 0.02	2.30 (2.13–2.36)	0.81 (0.81–0.82)	2.24 (2.20–2.43)	1.26 (1.24–1.34)
8.	Cu-CaP coating	3.3 (2.9–3.7)	15.0 (15.0–15.2)	60.0 (55.3–64.5)	34 ± 11 *n*_1_ = 18	2.41 * (2.18–2.87) *p*_1_ < 0.05	0.82 (0.82–0.83)	2.20 * (2.17–2.22) *p*_1_ < 0.05	1.26 (1.23–1.32)

Note: * statistical significance with the control group; *p*_1_-*p*_7_—statistically significant differences with the corresponded groups according to the Mann–Whitney U-test; *n*—the number of tested samples (wells of culturing plates); *n*_1_—the number of investigated cells.

**Table 4:** Results of a 24 h *S. aureus* culture in agar medium after 2 h pre-incubation with the 7-day extracts of the MAO coatings on Ti and Ti-40Nb substrates, Me (Q1-Q3).

No.	Groups, *n* = 3	Relative area of *S. aureus* Colonies per Total Area of Petri Dish (%)	
		Ti Substrate	Ti-40Nb Substrate
1.	0.9% NaCl (control 1)	28 (27–29)	
2.	RPMI-1640 (control 2)	43 ^1^ (41–45)	42 ^1^ (40–45)
3.	CaP coating	40 ^1^ (40–44)	45 ^1^ (43–46)
4.	Zn-CaP coating	26 ^2,3^ (25–27)	35 ^1−3^ (34–37)
5.	Cu-CaP coating	39 ^1−3^ (38–39)	34 ^1−3^ (33–35)

Note: *n*—number of Petri dishes studied in each group; 1—statistical differences with control group 1; 2—statistical differences with control group 2; 3—statistical differences with CaP coating according to Mann–Whitney U-test. Relative area (%) = (total area of *S. aureus* growth/total area of Petri dish) × 100%.

**Table 5:** Effect of the tested samples on the histological composition of bone tissue grown subcutaneously from the bone marrow during the ectopic osteogenesis test in mice.

Groups, *n* = 3	Tissue lamellae Properties		
	Incidence of Tissue Lamellae, %	Incidence of Bone Formation, %	Histological Composition
**Ti substrate**			
CaP coating	100	100	Bone with marrow (Figure 8a); bone lamellae (Figure 8b).
Zn-CaP coating	100	67	Bone lamellae (Figure 8c); connective tissue ossification (Figure 8d); connective tissue (not shown).
Cu-CaP coating	100	67	Bone with marrow (Figure 8e); bone lamellae (Figure 8f); connective tissue (not shown).
**Ti-40Nb substrate**			
CaP coating	100	67	Bone lamellae (Figure 9a); connective tissue ossification (Figure 9b); connective tissue (not shown).
Zn-CaP coating	100	67	Bone lamellae (Figure 9c); connective tissue ossification (Figure 9d); adipose, muscle and connective tissues (not shown).
Cu-CaP coating	100	67	Bone lamellae (Figure 9e); connective tissue ossification (Figure 9f); connective tissue (not shown).

Note: *n*—the number of tested samples.
